# Prevalence and risk factors associated with high somatic cell count in Chinese dairy herds

**DOI:** 10.3389/fvets.2022.967275

**Published:** 2022-11-02

**Authors:** Zhaoju Deng, Kun Wang, Chuang Xu, Jie Cao, Chong Ma

**Affiliations:** ^1^Department of Clinical Veterinary Medicine, College of Veterinary Medicine, China Agricultural University, Beijing, China; ^2^Institute of Cereal and Oil Crops, Hebei Academy of Agricultural and Forestry Sciences, Shijiazhuang, China

**Keywords:** subclinical mastitis, risk factors, dairy herd improvement, somatic cell count, prevalence

## Abstract

This study aimed to (1) estimate the prevalence of cow-level high somatic cell count (SCC) in Chinese dairy herds and (2) identify potential factors associated with cow- and herd-level SCC variables. The monthly data on dairy herd improvement were collected from a total of 131 dairy herds in 11 provinces in China in 2019. Mixed models were constructed using the cow composite milk SCC and the variance of cow SCC as dependent variables separately and parity, seasons, days in milk (DIM), herd size, and farm types (family-owned vs. company-owned) as fixed effects, accounting for the nested random herd and cow effect. We used negative binomial regression using herd-level SCC-related variables, namely, monthly proportion of high SCC, monthly proportion of new high SCC, monthly proportion of chronic high SCC, and monthly proportion of new chronic high SCC as dependent variables separately against seasons, herd size, and farm types with the random herd effect. The overall average prevalence of high SCCs for each month per farm was 0.26 (2.5–97.5% quantile: 0–0.56). Company-owned farms performed better in herd SCC management. Seasons were significantly associated with all the aforementioned variables, and summer and autumn were the seasons associated with worse outcomes in herd SCCs. This study is the first to assess high SCC in a large number of Chinese dairy herds, which is useful for farms to tailor the on-farm mastitis control programs in China.

## Introduction

Udder health management is of high importance in dairy farming (1). The common indicators of poor udder health are clinical and subclinical mastitis. Clinical mastitis is mostly diagnosed by the visual inspection of milk and udders for clinical symptoms, while the detection of subclinical mastitis relies on enumerating the somatic cell count (SCC) using the on-farm California mastitis test (2) or SCC sensors (3, 4) or *via* the bacteriologic culturing of milk samples in a laboratory (2).

The dairy herd improvement (DHI) program has long been used to monitor subclinical mastitis (5), and many research studies used it to estimate the prevalence of herd-level subclinical mastitis (6–8). The DHI program was founded in the 1990s in China; in recent decades, we have witnessed increased participation of farms in the program and more reliable data being generated from the program.

Herd size has significantly increased as compared with that in the last decade; the percentage of herds with more than 100 cows has increased from 20% (2008) to 60% (2018) (9). Small family-owned farms have ceased operations because of low production and environmental pressure in the last decade. According to the China Dairy Data Report (2020), the average lactational milk production per cow increased from 4,800 kg (2009) to 7,800 kg (2019) during the last decade (10). The average herd geometric mean SCC was comparable to that at farms in developed dairy industries (11–13). In addition to the improvement in milk production, the udder health status of Chinese dairy herds improved as compared to that in previous years (10).

Isolated attempts have been made to estimate the prevalence (or incidence) of (sub)clinical mastitis and the distribution of mastitis-causing pathogens in different regions in China. Gao et al. reported that the incidence of clinical mastitis was 3.3 cases per 100 cows every month, and the corresponding major mastitis-causing pathogens were environmental pathogens coexisting with a relatively low prevalence of contagious pathogens in clinical mastitis cases among Chinese dairy herds (14). According to Bi et al., the most prevalent mastitis pathogen species in bulk milk samples of Chinese dairy herds were *Streptococcus agalactiae, Streptococcus dysgalactiae, Trueperella pyogenes*, and *Staphylococcus aureus* (15).

Current research mostly focuses on clinical mastitis; however, mastitis-related expenses attributed to subclinical mastitis (48%) are higher than those associated with clinical mastitis (34%) (13). Therefore, controlling subclinical mastitis is more important. The SCC is a key indicator of subclinical mastitis. Therefore, this study aimed to (1) estimate the prevalence of a high SCC in cows and (2) identify the potential factors associated with a high SCC in Chinese dairy herds.

## Materials and methods

### Data collection

Dairy farms that participated in the DHI program and that we were acquainted with were approached. Farms were included in the study based on their willingness to participate in the study and their access to DHI data. Finally, the DHI data of 134 farms in 11 provinces in 2019 were collected.

### Data cleaning

A total of 924,970 records from 105,644 cows at 134 farms in 11 provinces were collected. These records were cleaned using the following steps:

1. Records without parity information were deleted (48 records from 48 cows at one farm).
2. Records with a milk yield < 1 kg were deleted (751 records from 695 cows at 51 farms in 10 provinces).
3. Records with a percentage of fat exceeding the range of 1.5–8 were deleted (20,801 records from 13,765 cows at 74 farms in 10 provinces).
4. Records without SCC were deleted (109 records from 107 cows in 22 herds from eight provinces).
5. Records with SCC exceeding the performance range (1–9,999 × 1,000 cells/mL) of the Fossomatic SCC measurement were deleted (49,390 records from 15,301 cows at 95 farms in 10 provinces) (16).
6. Records from days in milk (DIM) exceeding the range of 7–305 days were deleted (154,496 records from 48,808 cows at 134 farms in 11 provinces).
7. Records from cows that had undergone fewer than two consecutive DHI tests were deleted (27,245 records from 18,466 cows at 134 farms in 11 provinces).

A total of 672,078 records from 85,407 cows at 131 farms in 11 provinces were included in the final dataset after data cleaning.

### SCC variables

To identify factors associated with a high SCC, we used cow- and herd-level SCC variables derived from the cow composite milk SCC.

### Cow-level SCC variables

The cow composite milk SCC and the variance of cow SCC for each cow in 2019 were used as the cow-level SCC variables. The variance of cow SCC in 2019 was calculated as the variance of SCC for each cow.

### Herd-level SCC variables

We calculated the following herd-level SCC variables for each month: herd average SCC (SCC_av_), herd average variance of SCC (SCC_var_), herd average proportion of high SCC (HiSCC), herd average proportion of new high SCC (NHiSCC), herd average proportion of chronic high SCC (CHiSCC), and herd proportion of new chronic high SCC (NCHiSCC). The SCC_av_ was calculated as the herd geometric average of SCC for each month, followed by log_10_ transformation; the herd average variance of SCC was calculated in the same way as SCC_av_. The HiSCC was calculated as the herd average proportion of SCC more than or equal to 200,000 cells/mL for each month. The herd average proportion of new high SCC was calculated as the herd average proportion of new high SCC for each month. New high SCC cases were defined as records with SCC revealing more than or equal to 200,000 cells/mL on the current DHI test date and revealing fewer than 200,000 cells/mL on the preceding DHI test date. The herd average proportion of chronic high SCC was the herd average proportion of SCC with more than two consecutive SCCs revealing ≥200,000 cells/mL on all DHI test dates, and chronic high SCC was defined as more than two consecutive SCCs revealing ≥200,000 cells/mL. The herd proportion of new chronic high SCC was calculated as the herd average proportion of new chronic high SCC, followed by more than two consecutive SCCs revealing fewer than 200,000 cells/mL.

### Statistical analyses

#### Cow-level SCC variables

To identify factors associated with the cow composite milk SCC and the variance of cow SCC in 2019, a mixed model with the cow composite milk SCC (linear mixed model) and the variance of cow SCC (negative binomial mixed model) used as a dependent variable separately against parity (1, 2, 3, 4, ≥5), DIM (categorized as 7–40, 41–100,101–200, 200–305), season (spring, summer, autumn, winter), farm type (family-owned farm vs company-owned farm), and herd size, accounting for the random herd and cow effect (only random herd effect for the variance of cow SCC).

#### Herd-level SCC variables

Negative binomial mixed models were constructed by considering herd-level SCC variables as dependent variables and herd size, season, and farm type as independent variables, accounting for the random herd effect. Negative binomial mixed models were built using the glmer.nb function in the lme4 package version 1.1–26 (17), and model selection was performed using the backward stepwise method *via* the Akaike information criterion. Statistical significance was considered when the *P*-value was < 0.05 in a two-tailed test, and all analyses were performed using R version 4.0.2 (18).

## Results

The farms were mostly located in north China, which covers major milk production areas in China.

### Descriptive statistics

**Figure 1:**
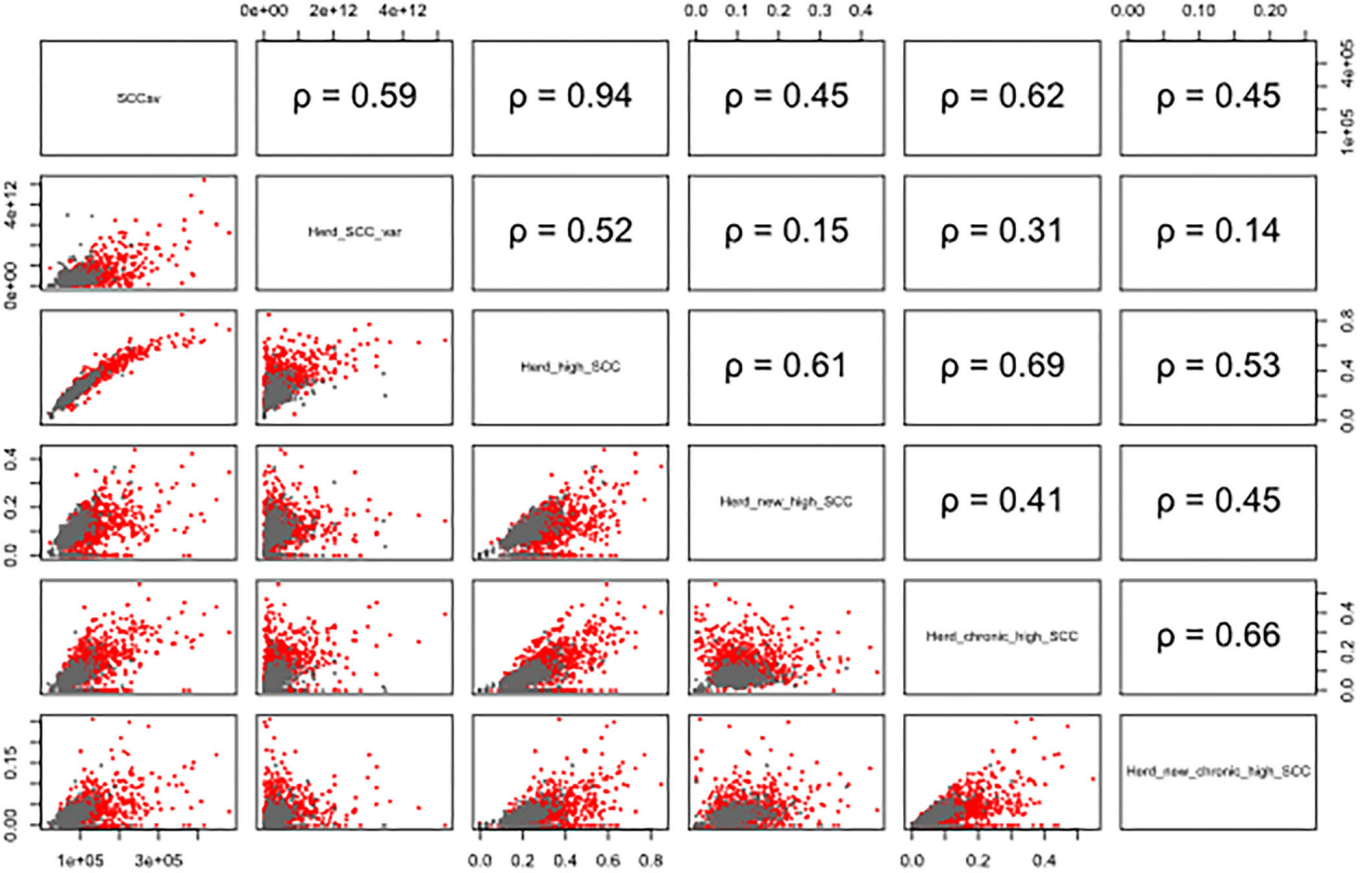
Scatterplot and the corresponding Pearson's correlation coefficient (ρ) among herd-level subclinical mastitis-related variables of the 131 farms in 11 provinces on a monthly basis. The gray points represent data from company-owned large herds, and red points show the data from family-owned small herds. Herd_SCC, monthly herd average of SCC; Herd_SCC_var, monthly variance of SCC within herd; Herd_high_SCC, monthly proportion of high SCC (>200,000 cells/mL); Herd_new_high_SCC, monthly proportion of new high SCC (SCC < 200,000 cells/mL in the previous test, while >200,000 cells/mL in the current DHI test); Herd_chronic_high_SCC, monthly proportion of chronic high SCC (>200,000 cells/mL in ≥2 consecutive DHI tests); Herd_new_chronic_high_SCC, monthly proportion of new chronic high SCC (SCC < 200,000 cells/mL in previous two consecutive DHI tests, >200,000 cells/mL in the current DHI test, and >200,000 in the following DHI test).

The overall average number of DHI tests per cow was 6.8 (2.5−97.5% quantile: 3–11), ranging from 5.8 (2.5−97.5% quantile: 3–10) to 7.8 (2.5−97.5% quantile: 3–11) per province. The overall average of daily milk yield per cow was 34.3 kg (2.5−97.5% quantile: 11.0–57.7), ranging from 30.2 (2.5−97.5% quantile: 12.6–51.5) to 38.4 (2.5-97.5% quantile: 10.4–75.4) per province. Detailed milk production data of each farm within each province are given in Table 1. Scatterplot and the corresponding Pearson's correlation coeffciient (r) among herd-level subclinical mastitis-related variables were illustrated in Figure 1.

**Table 1:** Descriptive statistics of the 131 farms in 11 provinces in 2019 in the final dataset.

**Province**	**Number of herds**	**Parity**	**Number of cows**	**Number of DHI tests per cow**	**Herd average milk yield per milking**	**Herd average of fat percentage per milking**	**Herd average of protein percentage per milking**
Beijing	22	Heifer	489 (101–1,427)	6 (2–10)	34.2 (28.2–38.9)	4.1 (3.4–4.7)	3.3 (3.1–3.4)
		Multiparous	797 (141–3,053)	6 (2–11)	38.4 (32.5–46)	4.2 (3.4–4.8)	3.3 (3.1–3.4)
Hebei	25	Heifer	169 (27–346)	6 (3–11)	27.1 (19.2–36)	4 (3.6–4.4)	3.4 (3.2–3.5)
		Multiparous	207 (28–396)	7 (2–11)	29.8 (21.8–38.4)	4 (3.6–4.4)	3.4 (3.2–3.6)
Heilongjiang	4	Heifer	597 (169–1,092)	5 (2–10)	31.7 (30.8–33.3)	3.6 (2.7–4.1)	3.3 (3.2–3.4)
		Multiparous	1,047 (381–2,049)	6 (2–11)	36.8 (34.5–39.1)	3.5 (2.6–4.1)	3.3 (3.3–3.4)
Henan	12	Heifer	455 (28–2,929)	7 (2–11)	27.5 (22.8–33.3)	3.9 (3.3–4.5)	3.4 (3.2–3.5)
		Multiparous	550 (80–3,071)	7 (1–11)	29.6 (25.2–37.4)	4 (3.3–4.5)	3.3 (3.2–3.4)
Hubei	1	Heifer	854 (854–854)	6 (3–9)	30.9 (30.9–30.9)	4.2 (4.2–4.2)	3.2 (3.2–3.2)
		Multiparous	1,055 (1,055–1,055)	6 (2–9)	35.8 (35.8–35.8)	4.5 (4.5–4.5)	3.3 (3.3–3.3)
Jiangsu	4	Heifer	1,887 (316–3,200)	6 (2–10)	31.5 (27.8–36.5)	3.7 (3.6–3.8)	3.2 (3.1–3.2)
		Multiparous	3,151 (357–5,976)	6 (2–10)	37.3 (34–40.3)	3.8 (3.7–3.9)	3.2 (3.2–3.3)
Shandong	2	Heifer	1,869 (867–2,871)	5 (2–9)	32.1 (31–33.2)	4 (3.9–4.1)	3.3 (3.3–3.3)
		Multiparous	2,454 (1,231–3,676)	5 (2–9)	38.1 (36.7–39.5)	4.2 (4.2–4.2)	3.3 (3.3–3.3)
Shanghai	8	Heifer	347 (224–626)	6 (2–10)	34.2 (30.5–36.5)	3 (2.6–3.5)	3.2 (3.1–3.2)
		Multiparous	519 (324–863)	7 (1–10)	37 (34.5–38.9)	3 (2.7–3.4)	3.2 (3.1–3.2)
Shanxi	42	Heifer	193 (59–362)	6 (2–10)	27.6 (23.2–33.5)	3.8 (3–4.4)	3.3 (3.2–3.6)
		Multiparous	277 (104–505)	7 (2–11)	30.7 (23.7–36.9)	3.8 (3.2–4.5)	3.3 (3.2–3.7)
Tianjin	10	Heifer	571 (230–998)	6 (2–10)	33.5 (29.4–36.9)	3.8 (3.2–4.4)	3.3 (3.2–3.4)
		Multiparous	694 (338–1,075)	6 (1–10)	38.8 (34.3–43.2)	3.8 (3.1–4.5)	3.3 (3.2–3.4)
Zhejiang	1	Heifer	736 (736–736)	6 (2–10)	27.3 (27.3–27.3)	4.6 (4.6–4.6)	3.4 (3.4–3.4)
		Multiparous	581 (581–581)	7 (1–11)	36.9 (36.9–36.9)	4.2 (4.2–4.2)	3.2 (3.2–3.2)

The median and 2.5–97.5% quantile are displayed for each variable.

The overall average monthly prevalence of high SCC was 0.26 (2.5–97.5% quantile: 0–0.59), ranging from 0.15 (2.5–97.5% quantile: 0.02–0.30) to 0.31 (2.5–97.5% quantile: 0–0.65) per province; the average prevalence values of high SCC in company-owned large herds and family-owned small herds were 0.21 (2.5−97.5%: 0.06–0.37) and 0.33 (2.5−97.5%: 0.14–0.60), respectively. Heifers exhibited fewer SCC-related variables than multiparous cows. The overall SCC_av_ of all farms was 109.9 (2.5–97.5% quantile: 39.3–271.0), ranging from 61.7 (2.5–97.5% quantile: 40.1–94.9) to 131.6 (2.5–97.5% quantile: 48.2–312.1) per province; the overall average of log_10_-transformed herd average variance of SCC was 11.4 (2.5–97.5% quantile: 10.0–12.3), ranging from 11.1 (2.5–97.5% quantile: 9.6–12.1) to 11.5 (2.5–97.5% quantile: 10.9–11.9) per province; the overall average of HiSCC was 0.26 (2.5–97.5% quantile: 0–0.59), ranging from 0.15 (2.5–97.5% quantile: 0.02–0.30) to 0.31 (2.5–97.5% quantile: 0–0.65) per province; the overall average of herd average proportion of new high SCC was 0.09 (2.5−97.5% quantile: 0–0.27), ranging from 0.05 (2.5−97.5% quantile: 0–0.10) to 0.12 (2.5−97.5% quantile: 0–0.34) per province; the overall average of herd average proportion of chronic high SCC was 0.09 (2.5–97.5% quantile: 0–0.32), ranging from 0.05 (2.5–97.5% quantile: 0–0.11) to 0.13 (2.5–97.5% quantile: 0–0.40) per province; and the overall average of herd proportion of new chronic high SCC was 0.03 (2.5−97.5% quantile: 0–0.11), ranging from 0.01 (2.5−97.5% quantile: 0–0.03) to 0.04 (2.5−97.5% quantile: 0–0.17) per province. The detailed descriptive statistics of SCC variables are provided in Table 2.

**Table 2:** Descriptive statistics of herd-level subclinical mastitis-related variables among 131 herds in 11 provinces in 2019.

**Province**	**Parity**	**Season**	**Herd SCC^a^**	**High SCC^b^**	**New high SCC^c^**	**Chronic high SCC^d^**	**New chronic SCC^e^**
Beijing	Heifer	Spring	64.55 (22.20–139.18)	0.14 (0.01–0.30)	0.08 (0–0.16)	0.04 (0–0.13)	0.02 (0–0.05)
		Summer	79.29 (26.52–134.76)	0.19 (0.02–0.36)	0.10 (0.01–0.23)	0.06 (0–0.17)	0.02 (0–0.06)
		Autumn	76.39 (23.26–121.50)	0.18 (0.01–0.33)	0.09 (0–0.19)	0.04 (0–0.14)	0.02 (0–0.06)
		Winter	72.40 (23.18–132.50)	0.16 (0.03–0.34)	0.06 (0–0.20)	0.03 (0–0.12)	0.01 (0–0.06)
	Multi parous	Spring	89.69 (29.56–155.50)	0.23 (0.03–0.40)	0.11 (0–0.19)	0.09 (0–0.24)	0.03 (0–0.07)
		Summer	107.5 (29.33–177.75)	0.27 (0.03–0.44)	0.13 (0.01–0.24)	0.10 (0–0.24)	0.04 (0–0.10)
		Autumn	93.33 (29.76–161.30)	0.24 (0.04–0.41)	0.09 (0.02–0.17)	0.07 (0–0.18)	0.02 (0–0.06)
		Winter	91.01 (34.65–158.64)	0.24 (0.07–0.40)	0.07 (0–0.17)	0.05 (0–0.19)	0.01 (0–0.03)
Hebei	Heifer	Spring	116.89 (63.46–199.60)	0.29 (0.13–0.50)	0.11 (0–0.27)	0.12 (0.01–0.38)	0.03 (0–0.10)
		Summer	123.89 (62.75–241.40)	0.33 (0.13–0.62)	0.16 (0.01–0.38)	0.12 (0.02–0.28)	0.05 (0–0.16)
		Autumn	135.57 (50.89–346.84)	0.33 (0.10–0.70)	0.13 (0–0.35)	0.15 (0.03–0.40)	0.05 (0–0.17)
		Winter	115.22 (58.50–210.90)	0.27 (0.09–0.51)	0.06 (0–0.29)	0.11 (0–0.40)	0.01 (0–0.10)
	Multi parous	Spring	134.65 (69.08–244.27)	0.34 (0.18–0.54)	0.14 (0–0.27)	0.16 (0–0.34)	0.04 (0–0.15)
		Summer	141.10 (72.42–283.54)	0.36 (0.17–0.65)	0.16 (0–0.39)	0.15 (0.04–0.29)	0.06 (0–0.16)
		Autumn	152.65 (73.03–347.45)	0.38 (0.18–0.76)	0.15 (0.04–0.33)	0.17 (0.01–0.44)	0.06 (0–0.19)
		Winter	133.37 (66.23–260.24)	0.34 (0.15–0.60)	0.06 (0–0.23)	0.14 (0–0.51)	0.03 (0–0.23)
Heilongjiang	Heifer	Spring	73.09 (64.21–88.30)	0.17 (0.10–0.28)	0.03 (0–0.09)	0.02 (0–0.04)	0 (0–0.01)
		Summer	64.18 (40.05–84.39)	0.17 (0.08–0.27)	0.09 (0.04–0.14)	0.03 (0.01–0.08)	0.01 (0–0.03)
		Autumn	80.36 (66.30–95.66)	0.22 (0.17–0.28)	0.06 (0–0.16)	0.07 (0–0.24)	0.03 (0–0.11)
		Winter	79.53 (67.18–100.03)	0.21 (0.12–0.28)	0.10 (0.01–0.18)	0.08 (0.01–0.17)	0.02 (0–0.04)
	Multi parous	Spring	94.00 (83.59–114.96)	0.26 (0.22–0.33)	0.05 (0–0.12)	0.05 (0–0.13)	0.01 (0–0.04)
		Summer	87.92 (63.07–115.59)	0.25 (0.19–0.33)	0.11 (0.06–0.16)	0.08 (0.05–0.13)	0.02 (0–0.04)
		Autumn	113.97 (93.21–144.07)	0.31 (0.28–0.36)	0.07 (0–0.16)	0.09 (0–0.27)	0.03 (0–0.09)
		Winter	109.23 (90.82–134.17)	0.30 (0.27–0.35)	0.12 (0.01–0.18)	0.11 (0.01–0.17)	0.02 (0–0.05)
Henan	Heifer	Spring	103.25 (44.33–260.03)	0.24 (0.04–0.63)	0.08 (0–0.32)	0.06 (0–0.21)	0.02 (0–0.11)
		Summer	119.91 (60.11–305.01)	0.29 (0.11–0.78)	0.12 (0.01–0.25)	0.11 (0–0.58)	0.03 (0–0.13)
		Autumn	119.45 (49.28–242.76)	0.28 (0.03–0.59)	0.14 (0–0.29)	0.10 (0–0.22)	0.04 (0–0.12)
		Winter	112.04 (36.17–254.09)	0.28 (0.03–0.68)	0.13 (0–0.52)	0.05 (0–0.22)	0.01 (0–0.07)
	Multi parous	Spring	108.59 (50.89–217.87)	0.28 (0.12–0.57)	0.08 (0–0.25)	0.07 (0–0.23)	0.01 (0–0.08)
		Summer	143.44 (63.28–403.28)	0.34 (0.16–0.74)	0.13 (0.01–0.36)	0.13 (0–0.38)	0.04 (0–0.12)
		Autumn	145.89 (69.39–344.70)	0.35 (0.17–0.60)	0.13 (0.04–0.24)	0.14 (0.01–0.38)	0.05 (0–0.13)
		Winter	119.31 (48.37–224.73)	0.32 (0.12–0.53)	0.09 (0–0.27)	0.09 (0–0.26)	0.02 (0–0.11)
Hubei	Heifer	Spring	39.29 (39.29–39.29)	0.05 (0.05–0.05)	0 (0–0)	0 (0–0)	0 (0–0)
		Summer	49.20 (41.79–53.33)	0.12 (0.08–0.14)	0.06 (0.04–0.08)	0.04 (0.02–0.05)	0.02 (0–0.03)
		Autumn	52.55 (48.27–56.67)	0.14 (0.12–0.15)	0.05 (0.04–0.06)	0.05 (0.05–0.06)	0.01 (0.01–0.02)
		Winter	45.30 (42.54–48.05)	0.08 (0.07–0.09)	0.02 (0–0.03)	0.01 (0–0.02)	0 (0–0)
	Multi parous	Spring	59.62 (59.62–59.62)	0.15 (0.15–0.15)	0 (0–0)	0 (0–0)	0 (0–0)
		Summer	83.24 (72.75–92.06)	0.23 (0.22–0.25)	0.10 (0.09–0.11)	0.08 (0.07–0.10)	0.02 (0–0.03)
		Autumn	87.01 (79.39–96.16)	0.27 (0.23–0.32)	0.08 (0.06–0.09)	0.10 (0.08–0.13)	0.03 (0.02–0.03)
		Winter	52.37 (52.02–52.72)	0.13 (0.13–0.13)	0.03 (0–0.06)	0.03 (0–0.05)	0 (0–0)
Jiangsu	Heifer	Spring	49.41 (38.58–63.47)	0.12 (0.09–0.20)	0.07 (0.05–0.12)	0.04 (0.01–0.08)	0.01 (0–0.04)
		Summer	61.06 (50.39–75.54)	0.16 (0.10–0.21)	0.08 (0–0.13)	0.04 (0–0.08)	0.02 (0–0.03)
		Autumn	62.11 (47.30–71.96)	0.17 (0.12–0.20)	0.08 (0.01–0.12)	0.04 (0–0.09)	0.02 (0–0.04)
		Winter	55.32 (48.19–67.72)	0.13 (0.11–0.15)	0.03 (0–0.09)	0.02 (0–0.06)	0 (0–0.01)
	Multi parous	Spring	68.50 (56.08–81.27)	0.20 (0.15–0.24)	0.10 (0.06–0.14)	0.07 (0.05–0.12)	0.02 (0–0.04)
		Summer	95.01 (66.44–153.18)	0.26 (0.17–0.39)	0.10 (0–0.24)	0.07 (0–0.12)	0.02 (0–0.05)
		Autumn	100.87 (75.11–141.52)	0.28 (0.24–0.35)	0.11 (0.01–0.17)	0.09 (0.01–0.16)	0.03 (0–0.07)
		Winter	66.10 (56.95–81.58)	0.19 (0.16–0.25)	0.04 (0–0.12)	0.04 (0–0.09)	0 (0–0.02)
Shandong	Heifer	Spring	55.88 (43.99–66.53)	0.13 (0.09–0.15)	0.04 (0–0.09)	0.02 (0–0.05)	0.01 (0–0.03)
		Summer	58.42 (42.83–75.93)	0.15 (0.09–0.24)	0.08 (0.04–0.12)	0.05 (0.02–0.12)	0.02 (0–0.07)
		Autumn	69.19 (60.56–75.70)	0.18 (0.13–0.23)	0.10 (0.05–0.12)	0.04 (0.02–0.06)	0.01 (0–0.02)
		Winter	66.24 (56.08–76.40)	0.14 (0.10–0.17)	0.06 (0–0.12)	0.02 (0–0.04)	0.01 (0–0.01)
	Multi parous	Spring	73.80 (60.70–81.42)	0.22 (0.16–0.26)	0.06 (0–0.11)	0.06 (0–0.14)	0.01 (0–0.04)
		Summer	82.55 (71.55–101.29)	0.24 (0.19–0.29)	0.11 (0.07–0.15)	0.09 (0.05–0.13)	0.03 (0–0.07)
		Autumn	87.21 (71.92–107.93)	0.25 (0.21–0.31)	0.10 (0.09–0.13)	0.08 (0.04–0.11)	0.02 (0–0.04)
		Winter	93.55 (79.95–107.15)	0.25 (0.22–0.28)	0.06 (0–0.12)	0.05 (0–0.10)	0.02 (0–0.04)
Shanghai	Heifer	Spring	52.97 (33.91–84.42)	0.13 (0.06–0.21)	0.07 (0–0.16)	0.03 (0–0.07)	0.01 (0–0.03)
		Summer	67.78 (42.31–134.61)	0.18 (0.09–0.37)	0.11 (0.04–0.26)	0.05 (0.01–0.1)	0.02 (0–0.08)
		Autumn	77.75 (47.90–137.23)	0.20 (0.12–0.39)	0.09 (0.02–0.21)	0.07 (0.01–0.13)	0.03 (0–0.09)
		Winter	58.33 (35.78–109.57)	0.15 (0.08–0.29)	0.06 (0–0.24)	0.02 (0–0.05)	0.01 (0–0.03)
	Multi parous	Spring	77.51 (59.22–93.71)	0.22 (0.17–0.27)	0.09 (0–0.14)	0.07 (0–0.11)	0.01 (0–0.04)
		Summer	102.34 (62.85–185.42)	0.28 (0.16–0.50)	0.14 (0.06–0.31)	0.11 (0.04–0.17)	0.04 (0.01–0.09)
		Autumn	119.89 (70.54–180.85)	0.32 (0.22–0.46)	0.11 (0.03–0.17)	0.13 (0.03–0.28)	0.04 (0–0.13)
		Winter	87.82 (54.47–147.35)	0.26 (0.17–0.37)	0.06 (0–0.19)	0.04 (0–0.10)	0.01 (0–0.04)
Shanxi	Heifer	Spring	150.19 (55.71–351.78)	0.34 (0.10–0.63)	0.11 (0.01–0.24)	0.14 (0–0.44)	0.04 (0–0.10)
		Summer	127.53 (44.07–328.39)	0.32 (0.14–0.56)	0.11 (0–0.29)	0.08 (0–0.28)	0.03 (0–0.10)
		Autumn	118.23 (42.46–271.90)	0.28 (0.10–0.53)	0.10 (0–0.26)	0.11 (0–0.30)	0.03 (0–0.09)
		Winter	107.66 (45.15–237.40)	0.26 (0.09–0.54)	0.08 (0–0.22)	0.09 (0–0.28)	0.02 (0–0.08)
	Multi parous	Spring	197.07 (76.07–422.60)	0.43 (0.22–0.67)	0.12 (0.06–0.22)	0.21 (0.05–0.38)	0.05 (0.01–0.11)
		Summer	169.54 (70.80–379.65)	0.39 (0.14–0.75)	0.13 (0.03–0.28)	0.14 (0.02–0.40)	0.04 (0–0.13)
		Autumn	156.69 (59.35–284.13)	0.38 (0.19–0.60)	0.11 (0.01–0.23)	0.17 (0.01–0.34)	0.04 (0–0.11)
		Winter	137.25 (61.03–309.86)	0.34 (0.16–0.60)	0.09 (0–0.22)	0.14 (0–0.36)	0.03 (0–0.11)
Tianjin	Heifer	Spring	47.05 (31.79–63.42)	0.12 (0.06–0.19)	0.06 (0–0.10)	0.03 (0–0.07)	0.01 (0–0.03)
		Summer	55.38 (27.67–110.55)	0.15 (0.03–0.32)	0.08 (0.01–0.22)	0.03 (0–0.08)	0.01 (0–0.03)
		Autumn	57.98 (37.77–87.53)	0.14 (0.06–0.22)	0.07 (0.02–0.16)	0.03 (0.01–0.06)	0.01 (0–0.03)
		Winter	50.11 (36.42–82.79)	0.11 (0.05–0.19)	0.04 (0–0.13)	0.02 (0–0.06)	0.01 (0–0.04)
	Multi parous	Spring	68.45 (38.86–109.18)	0.21 (0.11–0.33)	0.07 (0–0.13)	0.08 (0–0.20)	0.02 (0–0.04)
		Summer	72.06 (31.98–130.33)	0.23 (0.10–0.38)	0.10 (0.02–0.23)	0.07 (0–0.20)	0.02 (0–0.05)
		Autumn	78.77 (55.59–112.28)	0.23 (0.16–0.34)	0.09 (0.03–0.14)	0.08 (0.02–0.16)	0.02 (0–0.04)
		Winter	75.17 (49.96–112.32)	0.23 (0.14–0.32)	0.05 (0–0.15)	0.06 (0–0.16)	0.01 (0–0.04)
Zhejiang	Heifer	Spring	59.58 (58.68–60.48)	0.14 (0.13–0.14)	0.06 (0.05–0.08)	0.06 (0.04–0.08)	0.02 (0.01–0.02)
		Summer	47.92 (46.38–49.83)	0.11 (0.11–0.11)	0.05 (0.04–0.05)	0.05 (0.04–0.05)	0.02 (0.01–0.02)
		Autumn	65.90 (52.47–86.19)	0.16 (0.13–0.21)	0.07 (0.02–0.13)	0.05 (0.04–0.06)	0.01 (0.01–0.02)
		Winter	68.16 (58.54–74.29)	0.16 (0.13–0.17)	0.02 (0–0.05)	0.07 (0–0.13)	0.01 (0–0.020)
	Multi parous	Spring	85.47 (84.21–87.39)	0.23 (0.23–0.24)	0.08 (0.07–0.10)	0.12 (0.11–0.13)	0.03 (0.03–0.03)
		Summer	97.26 (89.78–102.96)	0.24 (0.23–0.25)	0.10 (0.08–0.11)	0.11 (0.09–0.12)	0.04 (0.03–0.04)
		Autumn	113.92 (105.70–120.03)	0.30 (0.29–0.31)	0.13 (0.11–0.15)	0.12 (0.11–0.14)	0.04 (0.03–0.05)
		Winter	97.36 (81.79–115.32)	0.28 (0.24–0.31)	0.03 (0–0.09)	0.14 (0.01–0.31)	0.04 (0–0.11)

The estimates represent the mean and corresponding 2.5–97.5% quantile for each variable.

a Monthly herd average of SCC.

b Monthly herd average proportion of high SCC (>200,000 cells/mL).

c Monthly herd average proportion of new high SCC (SCC < 200,000 cells/mL in the previous test, while >200,000 cells/mL in the current DHI test).

d Monthly herd average proportion of chronic high SCC (>200,000 cells/mL in ≥2 consecutive DHI tests).

e Monthly herd average proportion of new chronic high SCC (SCC < 200,000 cells/mL in previous two consecutive DHI tests, >200,000 cells/mL in the current DHI test, and >200,000 in the following DHI test).

### Factors associated with subclinical mastitis-related variables

#### Cow-level high SCC risk factors

The number of DHI tests was significantly negatively associated with the log_10_-transformed cow SCC. Parity was significantly positively associated with the cow SCC. The cow SCC decreased during 41–100 DIM and then increased on the following days as compared with that observed in the first 40 DIM. Season was significantly associated with the cow SCC; summer and autumn were the seasons with a high cow SCC as compared with spring. Variance of cow SCC was positively associated with parity, and cows in company-owned large herds had more stable SCCs than those in family-owned small herds. The estimates of each variable are listed in Table 3.

**Table 3:** Factors associated with cow-level high SCC among 131 herds in 11 provinces.

**Variable**	**Estimate (95% CI)**	
	**Cow SCC**	**Variance of cow SCC**
Intercept	5.05 (5.02–5.08)	13.97 (13.84–14.1)
Total number of DHI tests	−0.01 (−0.02–−0.01)	
Farm type		
Family farm	Ref	Ref
Company-owned farm	−0.22 (−0.26–−0.17)	−0.24 (−0.45–−0.04)
Parity		
1	Ref	Ref
2	0.08 (0.08–0.08)	0.28 (0.27–0.28)
3	0.13 (0.13–0.14)	0.42 (0.41–0.42)
4	0.18 (0.18–0.19)	0.52 (0.51–0.53)
≥5	0.22 (0.22–0.23)	0.59 (0.58–0.60)
Days in milk		
7–40	Ref	
41–100	−0.05 (−0.05–−0.04)	
101–200	0.01 (0.01–0.01)	
201–305	0.10 (0.09–0.10)	
Season		
Spring	Ref	
Summer	0.04 (0.04–0.04)	
Autumn	0.05 (0.05–0.06)	
Winter	0.02 (0.01–0.02)	

Mixed models were constructed using cow composite milk somatic cell count (SCC) and variance of cow SCC of each cow as the dependent variable separately, and parity, DIM, herd size, farm type, and total number of DHI tests (only applied for cow SCC) as independent variables, accounting for the nested random herd and cow effect (no random cow effect in modeling the variance of cow SCC).

#### Herd-level high SCC risk factors

Of the variables included in modeling (farm type, season, and herd size), farm type and season were significantly associated with herd-level SCC variables. Though, company-owned large herds performed better in terms of herd SCC-related variables. Meanwhile, the company-owned large herds were more stable in intramammary infection dynamics than the family-owned small herds, as indicated by new high SCC and variation in SCC. Milk in summer and autumn seasons had a higher SCC than milk in spring and winter. Detailed estimates of each herd-level SCC variables are provided in Table 4.

**Table 4:** Factors associated with herd-level SCC among 131 herds in 11 provinces.

**Variable**		**Estimate (95% CI)**			
	**Herd SCC^a^**	**High SCC^b^**	**New high SCC^c^**	**Chronic high SCC^d^**	**New chronic SCC^e^**
Intercept	5.12 (5.09–5.15)	0.29 (0.27–0.32)	0.13 (0.12–0.14)	0.15 (0.13–0.17)	0.04 (0.04–0.05)
**Farm type**					
Family-owned	Ref	Ref	Ref	Ref	Ref
Company-owned	−0.19 (−0.23–−0.14)	0.63 (0.56–0.7)	0.73 (0.66–0.8)	0.42 (0.36–0.5)	0.51 (0.45–0.57)
**Season**					
Spring	Ref	Ref	Ref	Ref	Ref
Summer	0.03 (0.02–0.04)	1.11 (1.07–1.15)	1.30 (1.14–1.48)	1.10 (0.97–1.25)	1.53 (1.28–1.82)
Autumn	0.05 (0.03–0.06)	1.14 (1.09–1.17)	1.21 (1.06–1.38)	1.19 (1.05–1.35)	1.68 (1.42–1.99)
Winter	0.01 (0–0.03)	1.05 (1.02–1.08)	0.75 (0.66–0.84)	0.81 (0.71–0.91)	0.78 (0.66–0.92)

The linear mixed model was applied using log_10_-transformed herd SCC for each month as the dependent variable, and herd size, farm type, and season as fixed effects, accounting for the random herd effect. Negative binomial mixed models were constructed using the rest of the herd-level SCC as dependent variables separately, and herd size, farm type, and season as fixed effects, accounting for the random herd effect. Independent variables significantly associated with herd-level SCCs are presented in this table.

a Monthly herd average of SCC.

b Monthly herd proportion of high SCC (>200,000 cells/mL).

c Monthly herd proportion of new high SCC (SCC < 200,000 cells/mL in the previous test, while >200,000 cells/mL in the current DHI test).

d Monthly herd proportion of chronic high SCC (>200,000 cells/mL in ≥2 consecutive DHI tests).

e Monthly herd proportion of new chronic high SCC (SCC < 200,000 cells/mL in previous two consecutive DHI tests, >200,000 cells/mL in the current DHI test, and >200,000 in the following DHI test).

## Discussion

Herds were included in this study based on the willingness of the farms and their access to the DHI data. Herds were mostly located in north China. The distribution of herd size was in line with that of the China Dairy Data Report (2020) (10); this indicates that the herd size in our study is representative of the current Chinese dairy herds. However, because the current DHI program provides free laboratory tests for farms, extremely large herds (a herd size with >10,000 cows) were not included in this study, while large herds did not participate in the DHI program possibly due to the cost of milk sampling and the constraints of the testing capacity of the DHI laboratories.

The overall prevalence of high SCC (defined as an SCC in a single DHI test of >200,000 cells/mL) for all farms was 0.26 (2.5−97.5% quantile: 0–0.56), which is lower than 0.34 (19) and 0.54 (20), but it was identical to that (0.26) recorded in another recent study (21). The data from the China Dairy Data Report (2020) also indicate decreasing herd SCCs in recent decades. The prevalence of a high SCC was comparable to that in developed dairy industries (22). The overall proportion of new high SCC was 0.11 (2.5−97.5%: 0–0.27), which was higher than that in developed dairy industries (23, 24). These results suggest that the overall performance of the presumptive subclinical mastitis status in Chinese herds has improved, while efforts are required to further improve the udder health status in Chinese dairy herds.

### Factors associated with subclinical mastitis

Udder health was better managed in company-owned large herds than in family-owned small herds, as indicated by the SCC variables. The estimated average cow SCC in large herds was 69,183 cells/mL, which was lower than that in family-owned small herds, 114,815 cells/mL. One possible explanation for this may be that large herds are managed in a modularized way by professionals. In addition, the dynamics of intramammary infections (indicated by the variance of cow SCC, new high SCC, and new chronic high SCC) were less in company-owned large herds than in family-owned small herds. This could possibly be because of dilutive effect of SCC infected cow in a large number of cows. As the proportion of large herds increased to 60% during the last decade (9), udder health management in large herds is gaining importance in China.

Season was significantly associated with cow composite milk SCC: the SCC was highest in summer, followed by autumn, indicating the detrimental effect of heat stress on udder health. Ferreira and De Vries found that high temperature and humidity were positively associated with a decrease in milk production and an increase in the herd SCC (25). The number of dairy farms in recent years in south China, which has higher temperatures and humidity (e.g., Guangdong Province, Hubei Province, Zhejiang Province, and Shanghai), than in north China (e.g., Beijing, Hebei Province, and Heilongjiang Province) is growing. Therefore, in addition to animal welfare considerations, heat stress in these areas should also be considered when designing udder health management programs.

Parity was positively associated with the cow SCC and the variance of cow SCC, indicating that multiparous cows are likely to be more susceptible to intramammary infections (26). The dilutive effect was also detected in our study as the cow SCC was estimated to be the lowest during 40–100 DIM. Cows undergoing more DHI tests tend to have a slightly lower SCC (Table 3), possibly due to a positive effect of active participation in the DHI program for improving udder health.

### Outlook and challenges in udder health management in Chinese dairy herds

The current status of high SCC in Chinese dairy herds is, to a moderate extent, comparable to that in developed dairy industries, while efforts are still needed to further reduce the prevalence of subclinical mastitis. However, the establishment of national databases in the DHI data collection that can facilitate data-driven decision-making is still in its early stages of development. We noticed that the DHI data quality remained a concern, and the tools to analyze these on-farm data to produce understandable and easy-to-implement measurements for farms were still lacking. The proportion of discontinuation of participation in DHI tests was relatively high; however, the underlying explanations are unclear.

In our view, as the DHI program is free for dairy farms, the major hurdles of non-participation in the DHI program are the lack of human labor for sample collection and milk sample processing capacity in the DHI laboratories. Therefore, an increase in the processing capacity of the laboratories is needed in addition to the motivation of farms to participate in the DHI program to facilitate data-driven decision-making in subclinical mastitis management.

## Conclusion

In this study, we aimed to estimate the prevalence of a high SCC in Chinese dairy herds and to identify the factors associated with it. The prevalence of a high SCC was estimated at 0.26 (2.5–97.5% quantile: 0–0.56). Heterogeneity in udder health was observed among different provinces. Seasons with high temperature and humidity were associated with worse outcomes in high SCC. Company-owned large farms performed better than family-owned small farms in SCC management. Efforts to motivate farms to participate in the DHI program and utilize the DHI data to assist on-farm udder health decision-making are also warranted.

## Data availability statement

The original contributions presented in the study are included in the article/supplementary material, further inquiries can be directed to the corresponding author/s.

## Ethics statement

Ethical review and approval was not required for the study of animals in accordance with the local legislation and institutional requirements.

## Author contributions

ZD, CX, JC, and CM: conceptualization. ZD: methodology, software, formal analysis, investigation, data curation, writing—original draft preparation, and visualization. KW, JC, and CM: resources. ZD, KW, JC, and CM: writing—review and editing. JC and CM: supervision. All authors have read and agreed to the published version of the manuscript.

## Conflict of interest

The authors declare that the research was conducted in the absence of any commercial or financial relationships that could be construed as a potential conflict of interest.

## Publisher's note

All claims expressed in this article are solely those of the authors and do not necessarily represent those of their affiliated organizations, or those of the publisher, the editors and the reviewers. Any product that may be evaluated in this article, or claim that may be made by its manufacturer, is not guaranteed or endorsed by the publisher.
